# New Insights on the Photodegradation of Caffeine in the Presence of Bio-Based Substances-Magnetic Iron Oxide Hybrid Nanomaterials

**DOI:** 10.3390/ma11071084

**Published:** 2018-06-26

**Authors:** Davide Palma, Alessandra Bianco Prevot, Marcello Brigante, Debora Fabbri, Giuliana Magnacca, Claire Richard, Gilles Mailhot, Roberto Nisticò

**Affiliations:** 1 Department of Chemistry, University of Torino, via P. Giuria 7, 10125 Torino, Italy; davide.palma@etu.uca.fr (D.P.); debora.fabbri@unito.it (D.F.); giuliana.magnacca@unito.it (G.M.); 2 CNRS, SIGMA Clermont, Institut de Chimie de Clermont-Ferrand, Université Clermont Auvergne, F-63000 Clermont-Ferrand, France; marcello.brigante@uca.fr (M.B.); claire.richard@uca.fr (C.R.); gilles.mailhot@uca.fr (G.M.); 3 NIS (Nanostructured Interfaces and Surfaces) Centre, Via P. Giuria 7, 10125 Torino, Italy; 4 Polytechnic of Torino, Department of Applied Science and Technology DISAT, C.so Duca Degli Abruzzi 24, 10129 Torino, Italy

**Keywords:** advanced oxidation processes, bio-based substances, hybrid nanomaterials, magnetic materials, photo Fenton, caffeine

## Abstract

The exploitation of organic waste as a source of bio-based substances to be used in environmental applications is gaining increasing interest. In the present research, compost-derived bio-based substances (BBS-Cs) were used to prepare hybrid magnetic nanoparticles (HMNPs) to be tested as an auxiliary in advanced oxidation processes. Hybrid magnetic nanoparticles can be indeed recovered at the end of the treatment and re-used in further water purification cycles. The research aimed to give new insights on the photodegradation of caffeine, chosen as marker of anthropogenic pollution in natural waters, and representative of the contaminants of emerging concern (CECs). Hybrid magnetic nanoparticles were synthetized starting from Fe(II) and Fe(III) salts and BBS-C aqueous solution, in alkali medium, via co-precipitation. Hybrid magnetic nanoparticles were characterized via X-ray diffraction (XRD), thermo-gravimetric analysis (TGA) and Fourier transform infrared (FTIR) spectroscopy. The effect of pH, added hydrogen peroxide, and dissolved oxygen on caffeine photodegradation in the presence of HMNPs was assessed. The results allow for the hypothesis that caffeine abatement can be obtained in the presence of HMNPs and hydrogen peroxide through a heterogeneous photo-Fenton mechanism. The role of hydroxyl radicals in the process was assessed examining the effect of a selective hydroxyl radical scavenger on the caffeine degradation kinetic.

## 1. Introduction

Polluted water treatment as well as urban bio-waste (UBW) management represent two challenging key issues that have to be faced worldwide. On one side, access to water has been recognized as a fundamental human right by the United Nations General Assembly [1], and “clean water and sanitation” is one of the United Nations Sustainable Development Goals [2,3]. Water scarcity, poor water quality, and inadequate sanitation negatively impact millions of people across the world every year. Moreover, the way water is used, which can mainly be described as “linear” (that is extraction upstream, disinfection treatment processes, use and then application of more expensive treatment processes before discharging it downstream) has numerous malfunctions that threaten the health of people and the environment. Therefore, there is a rising demand for exploring integrated water use and water treatment processes seeking for a transition to a more circular water management [4]. A peculiar aspect related to water quality is represented by the so-called contaminants of emerging concern (CECs)—xenobiotics detected at very low level in natural water bodies, since their recalcitrant behavior is to be abated in (waste)water treatment. Contaminants of emerging concern span a variety of chemicals comprising pharmaceuticals and personal care products (PPCPs), endocrine-disrupting compounds (EDCs), flame retardants (FRs), pesticides, and artificial sweeteners (ASWs) and their metabolites (see References [5,6,7] and references therein). Based on their harmfulness to human and environment health, there is an increasing commitment to find efficient processes for their abatement [8,9,10,11,12,13,14]. A CEC abatement approach that has been proposed by several research groups is represented by the so-called advanced oxidation processes (AOPs) that exploit the generation of highly reactive species (mainly HO· radicals) to induce the oxidative degradation of the organic pollutants, until their complete mineralization is attained. Among AOPs, great and increasing interest has been devoted to both Fenton and photo-Fenton processes, with a particular emphasis on the possibility to implement the process at a mild circumneutral pH, which is more compatible with natural water bodies [15,16,17,18,19,20].

On the other side, the increasing environmental concern of our society with respect to UBW fate has resulted in the need for developing sustainable processes able to decrease waste production or, alternatively, to valorize them for other uses. This latter approach fits with the biorefinery concept that is integrating different biomass conversion approaches to obtain fuels, power, heat, and value-added chemicals. Materials such as compost, anaerobic digestate, and organic residues from mechanical-biological treatment of UBW can be studied as biomass that could be used to feed a biorefinery [21]. It was previously demonstrated that with this approach, it is possible to extract from UBW, anaerobically and/or aerobically treated, bio-based substances (hereinafter BBS) featuring similarly to soil humic substances under the structural and physicochemical point of view [22,23]. Bio-based substances have been tested in several technological and environmental applications, including wastewater treatment [24,25,26].

Coupling UBW development and water treatment can therefore be considered an interesting way to “close the loop” in the transition from a linear to a circular economy [27].

We previously reported promising performances of BBS derived from compost as an auxiliary in the photodegradation of a group of CECs in the presence of iron and hydrogen peroxide (i.e., in a photo-Fenton process) [28,29]. In the presence of added BBS, it was possible to operate at milder pH conditions, compared to the ones suggested for photo-Fenton pollutant degradation [30].

A further step is represented by moving to a heterogeneous process, where BBS can be used as coating of magnetic nanoparticles made of magnetite/maghemite, containing iron useful for both Fenton and photo-Fenton reactions and suitable to be recovered and reused after water treatment [16,31]. Actually, magnetite/maghemite particles have been proposed for Fenton and photo-Fenton pollutant degradation with promising results (see Reference [32] and references therein). Nevertheless, these oxides undergo oxidation in natural atmosphere, yielding non-magnetic hematite, and the addition of organic coatings during the magnetite synthesis could also act as stabilizing barrier against iron (II) oxidation [13,33]. The addition of BBS was demonstrated to stabilize magnetite/maghemite nanoparticles [34], and in the presence of this kind of materials, encouraging preliminary results have been obtained in the photodegradation of caffeine [35,36]. Indeed, the hybrid BBS-coated magnetic nanoparticles (HMNPs) enhanced the caffeine photodegradation in the presence of added hydrogen peroxide.

In the present paper, more insights are given on the photodegradation of caffeine, a highly-used stimulant contained in drinking products (e.g., coffee, tea, caffeinated beverages) as well as in many pharmaceutical and personal care products. Its presence in the environment has been extensively studied; due to its environmental ubiquity caffeine has become a commonly used anthropogenic marker for water pollution, and it has been included among the contaminants of emerging concern (CECs). Even if caffeine has been traditionally accepted as posing a low risk to aquatic environments, it might grant attention; its mixture of toxicity effects, together with the ability of caffeine to bio-accumulate in the tissues of some aquatic species could represent a potential environmental risk. Thus, several studies have been devoted to assess the environmental hazards posed by caffeine [37].

Compost-derived bio-based substances (BBS-Cs) have been previously obtained from commercially composted urban biowastes [38] following a standard procedure for the humic acid isolation from soils; these composted biowastes are derived from urban public park trimmings and home gardening residues aged for more than 180 days [22]. After isolation, BBS-Cs have been characterized for their physico-chemical features [22]. Compost-derived bio-based substances have been chosen to prepare hybrid magnetic nanoparticles to be tested in the caffeine photodegradation process in order to increase the value of a material that is a product of a “virtuous” waste management approach, but, even if commercially available, does not have a market value suitable for compensating the cost of its production (including the initial separate waste collection). The effect of several experimental variables has been considered and assessed, and the reusability of HMNPs has been checked.

## 2. Materials and Methods

### 2.1. Materials

#### 2.1.1. BBS-C Isolation from the Green Compost

**Scheme 1:**
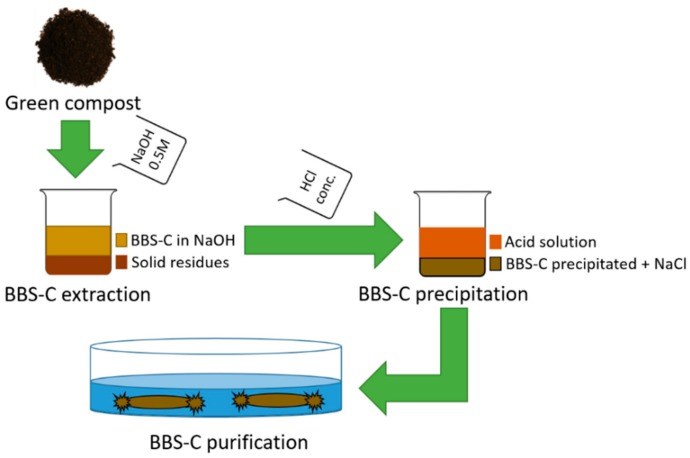
Schematic representation of compost-derived bio-based substance (BBS-C) isolation from compost.

Compost-derived bio-based substances were obtained from commercially-composted urban biowastes (*ACEA Pinerolese Industriale S.p.A.*, Pinerolo, Italy) [38]. The BBS-C isolation process was based on a standard procedure for the humic acid isolation from soils, and has been reported in Reference [22]. As shown in Scheme 1, it consists of a three-step process. In the first step, BBS-Cs were extracted from the green compost by alkaline washing with 0.5 M NaOH solution (1:10 solid–solution ratio, Sigma-Aldrich, Saint Louis, MO, USA) overnight in the dark. Then, the BBS-C solution was separated from the solid residues by several centrifugation and filtration steps (diameter: 2.7 μm). In the second step, BBS-Cs were precipitated via flocculation by varying the pH into acid (HCl conc. addition, pH < 1) and leaving the suspension overnight in the dark. Then the solid BBS-Cs were separated from the liquid phase by centrifugation (10,000 rpm, 20 min). In the last step, BBS-Cs were purified from NaCl residues (derived from the previous three steps) by cyclic washing with deionized water and subsequent dialysis in tubular membranes (cut-off 1 kDa) for 3–4 days rinsing with fresh-deionized water until the conductivity value decreases to 10 μS·cm^−1^. Finally, the suspension was freeze-dried, and solid BBS-C stocked.

#### 2.1.2. HMNPs Synthesis

The iron oxide magnetic nanoparticles were produced following a modified procedure based on a consolidated co-precipitation process reported in our previous studies [16,26,35,36,39]. In detail, 6.16 g FeCl_3_·6H_2_O (purity = 98%, CAS 10025-77-1, Sigma-Aldrich, Saint Louis, MO, USA) and 4.2 g FeSO_4_·7H_2_O (purity > 99.0%, CAS 7782-63-0, Sigma-Aldrich) were dissolved into 100 mL of deionized water. The solution was heated up to 90 °C under mechanical stirring. Afterwards, two solutions were added simultaneously: (i) 10 mL of an ammonia solution (30%, CAS 7664-41-7, Sigma-Aldrich), and (ii) 50 mL of a BBS-C solution containing the desired amount of BBS-C. Three different BBS-C solutions were investigated: 0.1, 0.2, and 1.0 wt.%. With respect to our previous work [36], smaller amounts of BBS-C were used to functionalize the magnetic material in order to evidence the effects of the experimental parameters examined and obtain information on material stability and reaction mechanisms. After 30 min at isothermal conditions (90 °C), nanoparticles obtained were magnetically separated using a commercial neodymium magnet, washing 5–6 times with fresh deionized water in order to remove the by-products of the reaction (i.e., ammonium salts). Finally, the BBS-Cs were deposited onto a glass Petri dish and dried at 80 °C overnight. Dried particles were crushed in a mortar. Samples thus obtained were named MH0.1, MH0.2, and MH1.0, depending on the BBS-C content in the solution used during the synthesis. Additionally, a reference material of pure magnetic iron oxide (coded M0) was synthesized in the absence of BBS-C solution.

#### 2.1.3. Other Reagents

Sodium hydroxide (NaOH, purity ≥ 98.0%, CAS 1310-73-2, Sigma-Aldrich), hydrochloric acid (HCl, conc. 37 wt.%, CAS 7647-01-0, Sigma-Aldrich), anhydrous potassium chloride (KCl, purity ≥ 99.0%, CAS 7447-40-7, Fluka), caffeine (CAS 58-08-2, Sigma-Aldrich), H_2_O_2_ (*v*/*v* 30%, CAS 7722-84-1, Sigma-Aldrich), *orto*-phenanthroline (purity 99.0%, CAS 66-71-7, Sigma-Aldrich), methanol (HPLC grade, ≥99.9%, CAS 67-56-1, Sigma-Aldrich), and 2-propanol (purity 95%, CAS 67-63-0, Sigma-Aldrich) were used throughout the work. All aqueous solutions were prepared using ultrapure water Millipore Milli-Q™ (Merk, Darmstadt, Germany). All chemicals were used without further purification.

### 2.2. Methods

#### 2.2.1. HMNPs Characterization

Thermo-gravimetric analyses (TGA) were performed on a TGA Q600 STD (TA Instruments, New Castle, DE, USA), working under either inert (nitrogen) or oxidant (air) atmospheres. The weight losses and the degradation profiles were evaluated by heating ca. 5–10 mg of each sample in an open alumina pan, applying a heating ramp from 30 to 800 °C (rate 10 °C·min^−1^).

X-ray diffraction (XRD) patterns were collected on the powdery samples using a PW3040/60 X’Pert PRO MPD diffractometer (PANalytical, Malvern, UK), working with a Cu anode source, at 45 kV and 40 mA in a Bragg–Brentano geometry with flat configuration. The Scherrer equation applied to the (311) iron oxide phase reflection was used to estimate the average size of the crystalline domains.

(1) $$\tau =\frac{K\lambda }{\beta \cos\theta }$$

where, τ is the average size of the crystalline domains (in nm), K is the shape factor (a constant value, for quasi-spherical domains is 0.9), λ is the X-ray wavelength (for Cu source is 0.154 nm), β is the line broadening at half of the maximum intensity after subtracting the line broadening (radians), and θ is the Bragg angle (radians).

Fourier transform infrared (FTIR) spectroscopy was carried out by using a Vector 22 spectrophotometer (Bruker, Billerica, MA, USA) in transmission mode, working with 128 scans and 4 cm^−1^ of resolution in the 4000–400 cm^−1^ range. The instrument has a Globar source and a DTGS detector. Spectra were analyzed by dispersing the samples in KBr (1:20 wt. ratio) and pressing them, forming a homogeneous pellet.

*Orto*-phenanthroline colorimetric tests were performed on the aqueous phase of a dispersion of MH1.0 HMNPs to evaluate the iron released by the material. Tests were performed on three different aqueous phases of MH1.0 dispersion (concentration 200 mg L^−1^) at three different pH values (3.0, 5.0, and 7.0). The pH was adjusted by adding either HCl or NaOH diluted solutions. Suspensions were mechanically shacked overnight in the dark. Then HMNPs were separated by applying an external magnetic field by means of a neodymium magnet. The three supernatants were filtered at 0.45 µm and analyzed in a double-beam T90+ UV–vis spectrometer (PG Instruments Ltd., Lutterworth, UK), in a quartz cuvette, slow speed mode at 1 nm resolution in the 200–800 nm range. The colorimetric test was performed as follows: 4 mL of the supernatant were added with 1 mL of 0.1% *w*/*v o*-phenanthroline aqueous solution and 1 mL of phosphate buffer (pH = 4). In the presence of Fe(II) ions, the orange-red ferrous-tris-*orto*-phenanthroline complex was formed (λ_max_ = 510 nm). The quantification was realized by means of an external calibration (i.e., Fe(II) standards concentration 0.1, 0.5, 1.0, and 5.0 mg·L^−1^). Additionally, in order to evaluate the total iron amount (i.e., Fe(II) + Fe(III)), a spatula tip of ascorbic acid was added to the supernatant prior to the colorimetric test; this way also Fe(III) was reduced to Fe(II) [40], allowing the determination of the total iron concentration.

The stability of the organic matter at the HMNPs’ surface was evaluated on MH0.2 sample (200 mg·L^−1^) put in contact for 2 h with either deionized water or a 1.5 × 10^−3^ M H_2_O_2_ solution in the dark or under irradiation (340 nm). The presence of BBS-C fragments released from the HMNPs has been evaluated by UV–Vis spectroscopy considering the absorbance at λ = 254 and 365 nm, values reported in the literature as characteristic for the humic acids [41], and at which the original BBS-C aqueous solution absorbs.

#### 2.2.2. Caffeine Irradiation Test and Analysis

All the irradiation experiments were performed in a cylindrical reactor equipped with 6 Philips TL D 15W/05 tubular lamps having emission spectrum from 300 to 475 nm with a maximum centered at 365 nm. Two-hundred mL of solution were placed in a cylindrical quartz reactor with 4.5 cm internal diameter and maintained under mechanical stirring during the irradiation. Caffeine concentration was fixed at 5 mg·L^−1^ while HMNPs and H_2_O_2_, when used, were added at a concentration of 200 mg·L^−1^ and 1.5 × 10^−3^ M, respectively.

Caffeine concentration during the irradiation experiments was monitored using a Waters ACQUITY UPLC system (Waters S.p.A., Sesto San Giovanni (MI), Italy) with a Nucleodur C_18_ column (100 mm × 2 mm × 1.8 µm), runtime was 6 min long, and eluents were acidic MilliQ water (0.1% phosphoric acid) and acetonitrile with a flow rate of 0.2 mL·min^−1^. A gradient raising the percentage of ACN from 40% to 70% during the run was applied. The caffeine quantification wavelength was 260 nm, and retention time was 3.6 min. Before analysis, every sample containing HMNPs were magnet-cleaned (in order to avoid the presence of the magnetic nanoparticles) and filtered through PTFE membranes with 0.45 µm cut-off. In the experiments where H_2_O_2_ was used, all samples were spiked with methanol with a volume ratio sample of MeOH equal to 1:1 in order to inactivate a Fenton reaction in dark conditions; the concentration values of caffeine were corrected according to the dilution.

## 3. Results

### 3.1. HMNPs Main Physico-Chemical Features

**Figure 1:**
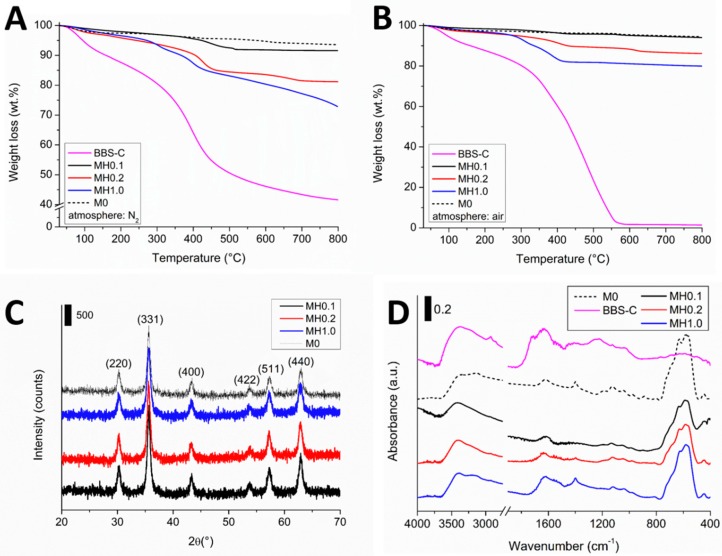
Physicochemical characterization of hybrid magnetic nanoparticles (HMNPs) and reference materials (BBS-C and M0): (**A**) thermo-gravimetric analysis (TGA) under nitrogen atmosphere, (**B**) TGA under air atmosphere; (**C**) X-ray diffraction (XRD) pattern of HMNPs (main reflections are labeled), and (**D**) absorbance FTIR spectra in transmission mode. Legend: M0 (black dotted line), BBS-C (magenta solid line), MH0.1 (black solid line), MH0.2 (red solid line), and MH1.0 (blue solid line).

The thermal stability and the organic matter content for all HMNPs were evaluated by TGA analyses performed under either reducing (nitrogen) or oxidant atmospheres (see Figure 1A,B, respectively). The thermal profiles obtained under a nitrogen atmosphere (Figure 1A) shows a first weak weight loss at ca. 100 °C due to the water molecules being physically sorbed at the surface, followed by a complex one in the 150–500 °C range corresponding to the pyrolysis of BBS-C, organized in a first contribution centered at 310 °C (only MH1.0) attributable to the carbohydrate residues, and the main one centered at ca. 430 °C due to the pyrolysis of BBS-C humic-like fraction, before forming a carbonaceous residue (BBS-C residue at 800 °C is ca. 40 wt.%) [35]. Once the pyrolysis temperature became higher than 700 °C, a weak weight variation was also registered for both the reference M0 and the HMNPs due to the iron oxide reduction to wustite (FeO) and elemental iron (Fe), in accordance with our previous studies [26,31]. When HMNPs were heated under an oxidant atmosphere (Figure 1B), three weight losses were registered: the first one (at ca. 100 °C), again due to the moisture content in the samples, whereas the main relevant one was comprised in the 250–400 °C range, and it was due to the oxidation of BBS-C to CO_2_ and other volatile products. This is since the atmosphere of oxidant BBS-C when thermally treated is completely mineralized leaving almost no residue at 600 °C. At ca. 580 °C (i.e., when the organic matter has been completely oxidized), a phase transformation from magnetite/maghemite to hematite started, thus leaving a reddish non-magnetic residue. Weight losses (calculated in the 150–500 °C range) associated with the organic matter content in the HMNPs were 2 wt.% for MH0.1, 7wt.% for MH0.2, and 15 wt.% for MH1.0, thus suggesting that higher BBS-C concentration during synthesis caused higher BBS-C loading in the nanomaterials.

The X-ray diffraction (Figure 1C) patterns for all HMNPs confirmed the crystalline reflections at 2θ = 30.1° (220), 35.4° (311), 43.0° (400), 53.9° (422), 57.2° (511), and 62.6° (440), consistent with the signals of the reference magnetite/maghemite M0 (card numbers 00-019-0629 and 00-039-1346, ICCD Database) [13,35]. The two phases (namely, magnetite and maghemite) cannot be distinguished by means of XRD, since both phases have the same identical XRD pattern. The only difference is that maghemite is the (topotactic) oxidized form of magnetite, and it is a common procedure to consider the simultaneous presence of both phases [13,35]. Applying the Scherrer equation on the main reflection peak at (311), it is possible to estimate the average size of the crystalline domains, being in the 16–18 nm range for all HMNPs. No interesting peaks were registered for BBS-C since it was amorphous. The exact composition of the BBS-C is very difficult to clarify due to the high complexity of humic substances. As reported in our previous work [22], these BBS-Cs are supramolecular aggregates made by macromolecules containing aromatic and aliphatic chains with lots of functionalities (mostly, O-containing groups). On the basis of the FTIR analysis reported in Figure 1D, the main signals of BBS-C are the OH stretching mode at 3400 cm^−1^, the CH stretching vibrations in the 3000–2800 cm^−1^, the C=O stretching mode of the carbonyl functionalities at ca. 1740–1700 cm^−1^, the signal at ca. 630–550 cm^−1^ due to the Fe-O vibration mode of both the octahedral and tetrahedral sites forming the magnetite/maghemite iron oxide, and several broad signals in the 1650–1560 cm^−1^ range due to C=C vibrations [36,41]. The attribution of the bands at 1400 cm^−1^ deserves more attention. Indeed, significant differences in the spectra bands related to carboxylic and carboxylate groups can be observed between the free BBS-C, M0, and hybrid MH samples. In particular, the pointed peak at 1400 cm^−1^ observed for the MH1.0 sample is consistent with a carboxylate–iron stretching, as previously reported [35,36]. This characteristic signal decreases in the MH0.2 spectrum, and is not detectable in the MH0.1 spectrum. Nevertheless, we have to take into account that the organic matter amount decreases going from MH1.0 to MH0.1, and the absence of the signal ascribed to the BBS-O-Fe could be just a matter of detection limit. An alternative hypothesis could be that the signal at 1400 cm^−1^ was due to the CO bond stretching mode of the carbonate groups formed from the atmospheric CO_2_; nevertheless, we discarded this latter hypothesis since the signal intensity should not vary among the different HMNPs.

Reference M0 shows the main signal at ca. 630–550 cm^−1^ (see comment above). On the basis of the FTIR analysis, all HMNPs present the signals due to iron oxide (Fe-O bands), and some peaks attributable to BBS-C-derived organic matter at the surface forming the HMNPs [35].

### 3.2. HMNPs Stability

In order to evaluate the stability of the HMNPs systems, spectroscopic analyses were performed to quantify the iron released in water (at different pH values), and the release of BBS-C at the experimental conditions. Concerning the release of both Fe(II) and Fe(III) ions in water, a suspension of MH1.0 (200 mg·L^−1^) was taken as reference for all the HMNPs. A colorimetric test was performed (see Section 2.2.1) after 12 h of HMNP dispersion in ultrapure water. Three different pH conditions were investigated, namely, pH = 3.0, 5.0, and 7.0. Results obtained evidenced that at strong acid pH (pH = 3.0) there was a significant release of Fe(II) from the iron oxide (Table 1). This effect is in agreement with the literature since magnetite/maghemite are sensible to strong acid [42]. On the contrary, at pH 5.0 and 7.0, a significantly higher stability of the materials, in terms of iron release, was observed.

**Figure 2:**
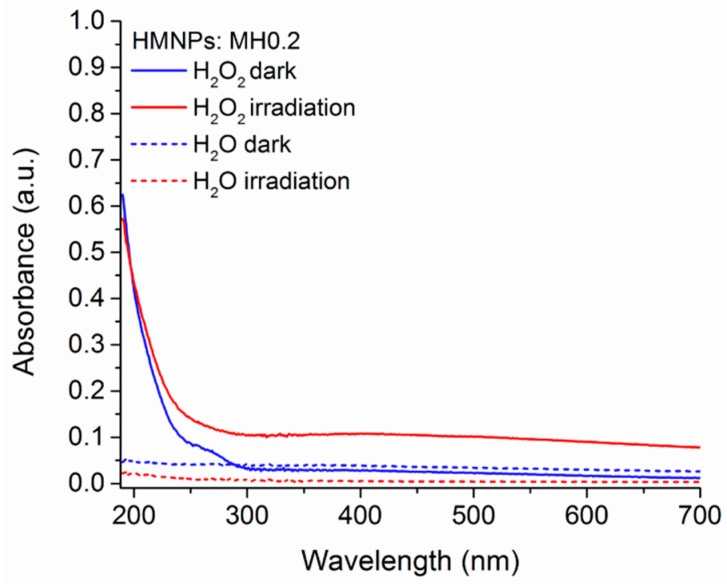
HMNP stability in solution: UV–Vis spectra of either water (dotted line) or 1.5 × 10^−3^ M H_2_O_2_ (solid line) solutions after being in contact with MH0.2 for 2 h either in the dark (blue) or under irradiation (red).

The eventual release of organic matter from HMNPs in solution was evaluated by measuring the UV–Vis spectra of the aqueous phase of MH0.2 (200 mg·L^−1^) suspensions after 2 h (either in the dark or upon light irradiation) with ultrapure water or with a 1.5 × 10^−3^ M H_2_O_2_ solution. As reported in Figure 2, neither the aqueous phase from the irradiated MH0.2 suspension, nor the one from the suspension left in the dark, showed any significant signals in the 190–700 nm wavelength range. Conversely, for the suspensions added by H_2_O_2_, the corresponding aqueous phases presented an absorption band at λ < 300 nm assignable to BBS-C (and/or its fragments) released from the HMNPs upon chemical oxidation (in the dark) and/or photochemical oxidation (under irradiation). The very slow but not negligible photodegradation of BBS was reported in a previous study, on the BBS photostability in a homogeneous system [43].

### 3.3. Preliminary Test on Caffeine (Photo)stability

The (photo)stability of caffeine has been preliminarily assessed in homogeneous systems by irradiating an aqueous solution at different experimental conditions: (i) without any additional substance, (ii) in the presence of 1.5 × 10^−3^ M added H_2_O_2_, and (iii) in the presence of 6 mg·L^−1^ of BBS-C (theoretical concentration corresponding to BBS-C immobilized on the HMNPs, estimated by TGA analysis). Tests (ii) and (iii) were performed also in the dark.

**Figure 3:**
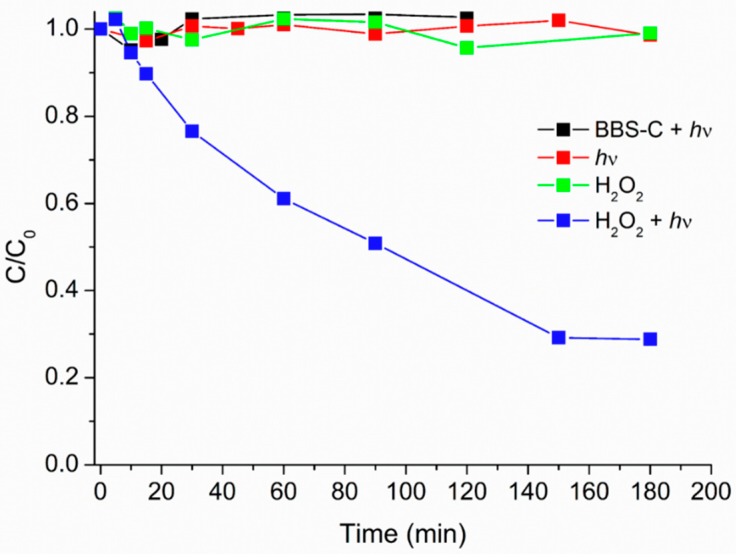
Caffeine concentration (C_0_ = 5 mg·L^−1^) profile in the presence of H_2_O_2_ (1.5 × 10^−3^ M) in dark condition (green squares); under irradiation (red squares); in the presence of BBS-C (6 mg·L^−1^), and under irradiation (black squares); in the presence of H_2_O_2_ (1.5 × 10^−3^ M), and under irradiation (blue squares).

From data reported in Figure 3, it can be observed that caffeine was not degraded in pure water, and also upon addition of BBS-C the caffeine abatement was negligible. As for this latter experiment, it must be noted that a very low amount of BBS-C was added, compared to the values reported in the literature in other applications of BBS to organic pollutant degradation [24,35].

In the presence of hydrogen peroxide 1.5 × 10^−3^ M, the caffeine concentration decreased by about 75% after 120 min under irradiation (Figure 3). Despite no degradation in the dark, the fast degradation under irradiation observed in the presence of hydrogen peroxide is attributed to the generation of hydroxyl radicals through the hydrogen peroxide photolysis.

### 3.4. Heterogeneous Caffeine (Photo)degradation in the Presence of HMNPs

Several preliminary experiments (data not shown) were performed on caffeine aqueous solution using the three synthesized HMNPs: (i) caffeine adsorption on the HMNPs, in the dark and (ii) caffeine degradation under irradiation. All the experiments were run for 180 min and the three HMNPs did not show significantly different behaviors; in both cases (i) and (ii), the caffeine disappearance was negligible. Different results were obtained when adding 1.50 × 10^−3^ M of H_2_O_2_ either in the dark or under light irradiation. The first phenomenon worth mentioning is the so-called “time zero”, that is a sample containing all the reagents, filtered immediately after being prepared, and then analyzed. It gave an initial concentration of caffeine from ca. 15 to 25% lower than the theoretic amount (5 mg·L^−1^), suggesting some H_2_O_2_ participation to the phenomenon in terms of Fenton reaction. One possible explanation can be correlated with a not perfectly controlled MH0.2 synthetic procedure, in particular it could be ascribed to the unknown hydration extent of FeCl_3_ used as source of Fe(III) in the synthesis of magnetite; as a result part of the Fe cations can be excluded from the crystalline framework of the magnetite/maghemite phase and affect the reactivity of the prepared material [16]. Indeed this amount of Fe(III) can be more easily available for forming photoactive complexes with the BBS-C moiety, thus justifying an initial very fast caffeine degradation

**Figure 4:**
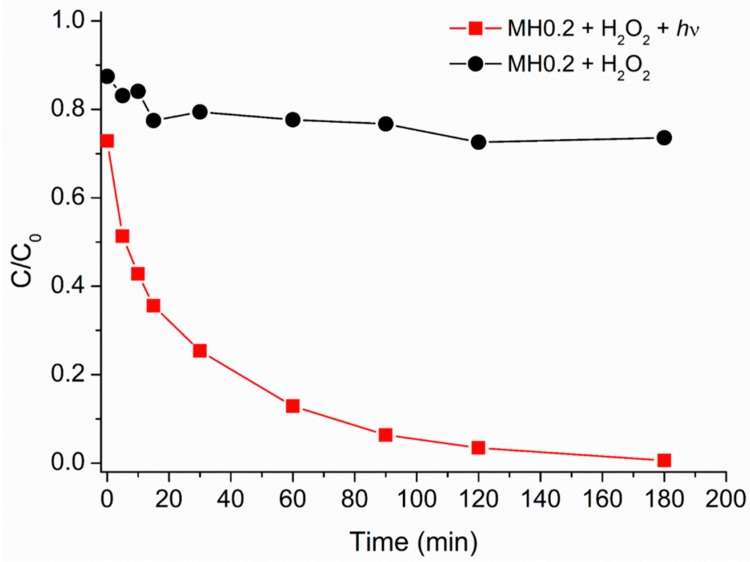
Caffeine relative degradation vs. irradiation time in the presence of MH0.2 (200 mg·L^−1^) and H_2_O_2_ (1.50 × 10^−3^ M). Data reported considering an initial caffeine concentration of 5 mg·L^−1^. Dark condition (black circles), light irradiation (red squares).

Moreover, the three HNMPs showed different efficiencies and the process was in all cases faster under irradiation. A test was performed in order to compare the efficiency of the three HMNPs: an aqueous solution of caffeine (5 mg·L^−1^) was irradiated for 30 min in the presence of HMNPs (200 mg·L^−1^) and H_2_O_2_ (1.50 × 10^−3^ M). The caffeine abatement in the presence of MH0.1, MH0.2, and MH1.0 was 19.5%, 65.0%, and 24.5%, respectively. Based on these results, MH0.2 was used throughout the rest of the work. Figure 4 shows the degradation profiles obtained in the presence of MH0.2 and hydrogen peroxide, either in the dark or under irradiation. As can be observed, the heterogeneous Fenton process yielded slight caffeine abatement in the first 30 min of contact and then remains stable, with an overall caffeine abatement of ca. 20%.

Based on these results no more tests were performed in the dark.

**Figure 5:**
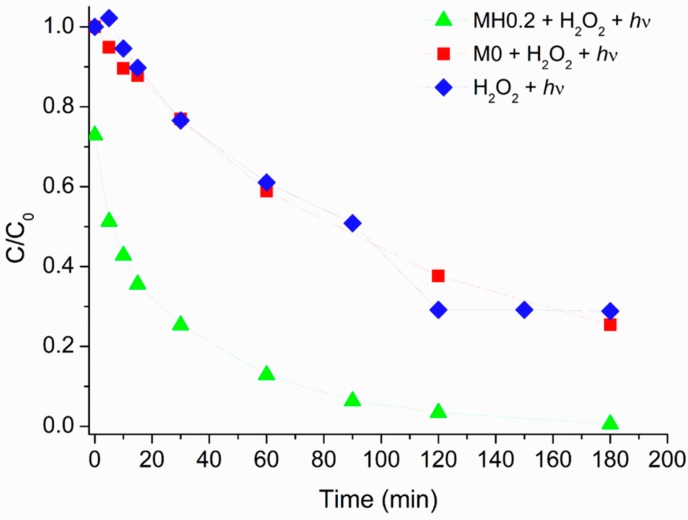
Caffeine relative photodegradation vs. irradiation time in the presence of: H_2_O_2_ (1.50 × 10^−3^ M) (blue diamonds); bare magnetite (M0, 200 mg·L^−1^) and H_2_O_2_ (1.50 × 10^−3^ M) (red squares); MH0.2 (200 mg·L^−1^) and H_2_O_2_ (1.50 × 10^−3^ M) (green triangles). Data reported considering an initial caffeine concentration of 5 mg·L^−1^.

The caffeine degradation profile obtained under irradiation in the presence of MH0.2 and H_2_O_2_ was then compared with data obtained by irradiating an aqueous caffeine solution in the presence of (i) bare magnetite (M0) and H_2_O_2_ 1.50 × 10^−3^ M, or (ii) H_2_O_2_ 1.50 × 10^−3^ M alone. As can be seen in Figure 5, in cases (i) and (ii), almost the same kinetic was obtained, allowing to hypothesize that the degradation process was mainly driven by the photolysis of H_2_O_2_ yielding hydroxyl radicals (HO·) responsible for the caffeine degradation. On the other side, the beneficial effect of having MH0.2 instead of bare magnetite clearly appears, thus allowing to envisage an active role of the organic moiety.

### 3.5. Effect of pH on the Photo-Fenton Caffeine Degradation Mediated by HMNPs

**Figure 6:**
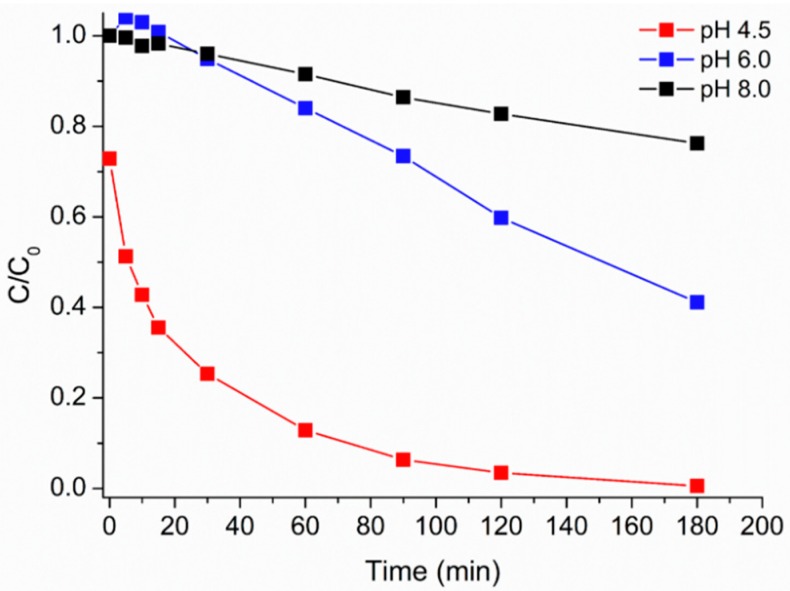
Caffeine relative photodegradation vs. irradiation time at different initial pH values: 4.5 (red squares), 6.0 (blue squares), and 8.0 (black squares) MH0.2 200 mg·L^−1^, H_2_O_2_ 1.50 × 10^−3^ M. Data reported considering an initial caffeine concentration of 5 mg·L^−1^.

Since it has been reported that photo-Fenton (and related) processes have their optimum pH at about 2.8, the effect of this experimental variable was checked, aiming to raise this value toward neutrality. We started from pH = 4.5, which was the value measured in the suspension of MH0.2 (200 mg·L^−1^); such acidic pH could be due to the presence of some hydroxo-Fe(III)-containing phase inside the iron-based MH0.2 core, resulting in Fe(III) hydrolysis. Different experiments were performed rising the initial pH value up to pH = 8.0. As it can be observed in Figure 6, also in the present case the best results could be obtained at lower pH; nevertheless, caffeine degradation was still achievable at pH 6.0 while its degradation rate became too slow at pH 8.0. It is worth to be noted that only at pH 4.5, the concentration at “zero time” was significantly lower than the theoric one. This behavior allows to consider soluble iron, together with the H_2_O_2_ previously mentioned, responsible for the initial degradation of the caffeine. In fact, only at a very acidic pH can iron species released from the material remain available in solution, whereas at a higher pH they could precipitate in the form of hydroxides.

### 3.6. Effect of Hydroxyl Radical Scavenger and Dissolved Oxygen on the Caffeine Photodegradation Mediated by HMNPs

**Figure 7:**
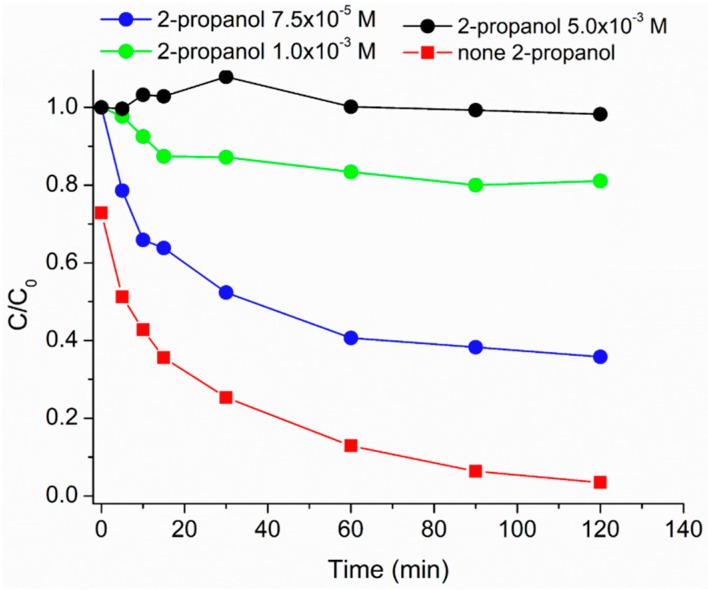
Caffeine relative photodegradation vs. irradiation time in the presence of different [2-propanol]: none 2-propanol (red squares), 7.5 × 10^−5^ M (blue circles), 1.0 × 10^−3^ M (green circles), and 5.0 × 10^−3^ (black circles). MH0.2 200 mg·L^−1^, H_2_O_2_ 1.50 × 10^−3^ M. Data reported considering an initial caffeine concentration of 5 mg L^−1^.

In order to get insight in the caffeine photodegradation mechanism, experiments have been performed in the presence of 2-propanol that is a HO·scavenger [44]; increasing amounts were added (i.e., 7.5 × 10^−5^ M, 1.0 × 10^−3^ M, and 5.0 × 10^−3^ M), and chosen in order to have a theoric inhibition of caffeine degradation of 50%, 97%, and 100%, respectively. The results reported in Figure 7 show a progressive inhibition effect of 2-propanol on caffeine photodegradation, even if the inhibition did not reach the expected extent in the first two cases. Nevertheless, the main role of HO· in the degradation process was clearly shown. Moreover experiments have been performed by controlling the reaction atmosphere by saturating the system with oxygen or with argon (data not shown); in the absence of oxygen, the degradation slowed down and the calculated first-order kinetic constant decreased from 0.0293 min^−1^ (in air/oxygen) to 0.0164 min^−1^ (in argon-saturated medium), thus confirming the predominant role of oxygenated reactive species as drivers of the caffeine degradation process.

### 3.7. Re-Use of HMNPs

**Figure 8:**
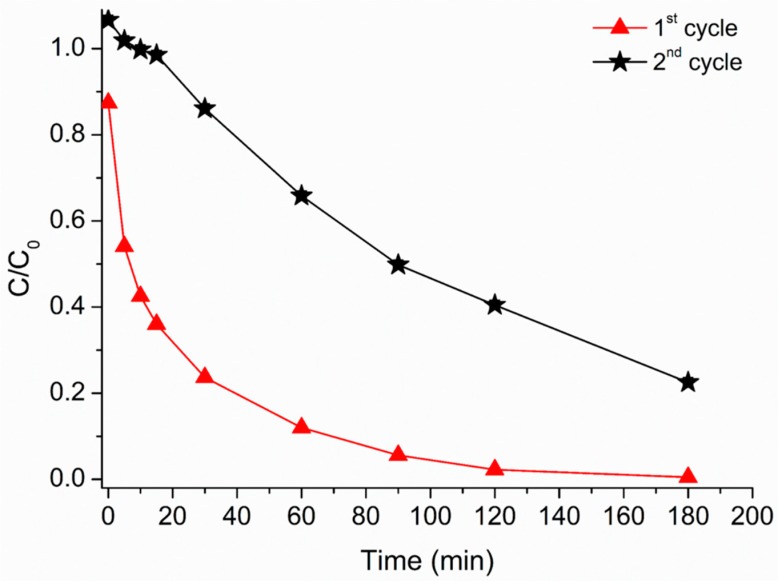
Caffeine relative concentration vs. irradiation time. 1st cycle (red triangles); 2nd cycle (black stars). MH0.2 200 mg·L^−1^, H_2_O_2_ 1.50 × 10^−3^ M. Data reported considering an initial caffeine concentration of 5 mg·L^−1^.

In order to consider the possibility to apply HNMPs in real wastewater treatment, an essay of recovery and reuse of MH0.2 was performed. After the first 180 min contact with caffeine under irradiation, MH0.2 were magnetically separated from the aqueous phase; further on, a fresh caffeine solution was added together with hydrogen peroxide and then submitted to irradiation for 180 min. Figure 8 shows the obtained results.

Several remarkable features could be observed: (i) an overall decrease in the caffeine degradation rate from the first to the second cycle, (ii) an initial 20 min of “induction” step during the second cycle while in the first one a very fast caffeine decay took place in the first 2 min, (iii) the “time zero” effect is visible only in the first cycle.

## 4. Discussion

The obtained results can be discussed by considering previous data reported in the literature on the use of magnetite as catalyst for photo-Fenton-like pollutant degradation. The negligible caffeine degradation observed under Fenton-like conditions is in agreement with the results reported for phenol degradation in the presence of different magnetite samples [32] and for Bisphenol A degradation in the presence of magnetite and ethylenediamine-*N*,*N*′-disuccinic acid (EDDS) [45], where the irradiation of magnetite with H_2_O_2_ was required to trigger reactivity. In the present work, the need of light to induce caffeine transformation and the fact that the relevance of irradiation was not related only to the photolysis of H_2_O_2_ allows for the hypothesis that Fe(III) photoreduction to Fe(II) was required to trigger the degradation process. Moreover, the enhanced efficiency observed when comparing MH0.2 towards photo-Fenton-like caffeine abatement with data obtained using bare magnetite was attributed to the presence of the organic BBS-C shell. A first reason could be that the organic shell, behaving as surfactant, enhanced the particle dispersion, yielding higher solid–liquid interface and higher reactivity. Hence, as demonstrated by the effect of added 2-propanol, hydroxyl radicals are mostly responsible for MH0.2 activity, and such species being highly reactive, it is reasonable to hypothesize that the caffeine degradation takes place at the nanoparticles-solution interface rather than in the bulk. A second reason could be that the organic moiety is able to form photoactive complexes with iron, in analogy with the findings (and related interpretation) of Huang and co-workers [45]. The authors stated that EDDS, as a strong chelating agent, maintained iron in soluble form, through Fe-EDDS complex, which is responsible for the superoxide radical anion formation, able to enhance the generation of Fe(II) (the rate-limiting step), and therefore the production of HO**·**. This implies the consideration also in the present work of the possible role of superoxide anion. In this context, BBS-C that should be able to generate reactive species under light excitation, as shown for other humic-like substances extracted from compost, could contribute to the formation of HO**·** and/or superoxide radical anion [46,47].

On the other side, a contribution to caffeine photodegradation related to iron and organic matter released in solution (even if at very low concentration), yielding a homogeneous photo-Fenton process cannot be ruled out. In fact, the sample as prepared and used in the photo-Fenton reaction shows the “time zero” phenomenon and a higher activity with respect to the activity of the sample recovered and reused (Figure 8). This behavior could be explained considering that the hydroxo-Fe(III)-containing phase previously mentioned causes the decrease of the pH for hydrolysis, giving soluble iron species active in homogeneous photo-Fenton process for caffeine abatement.

**Scheme 2:**
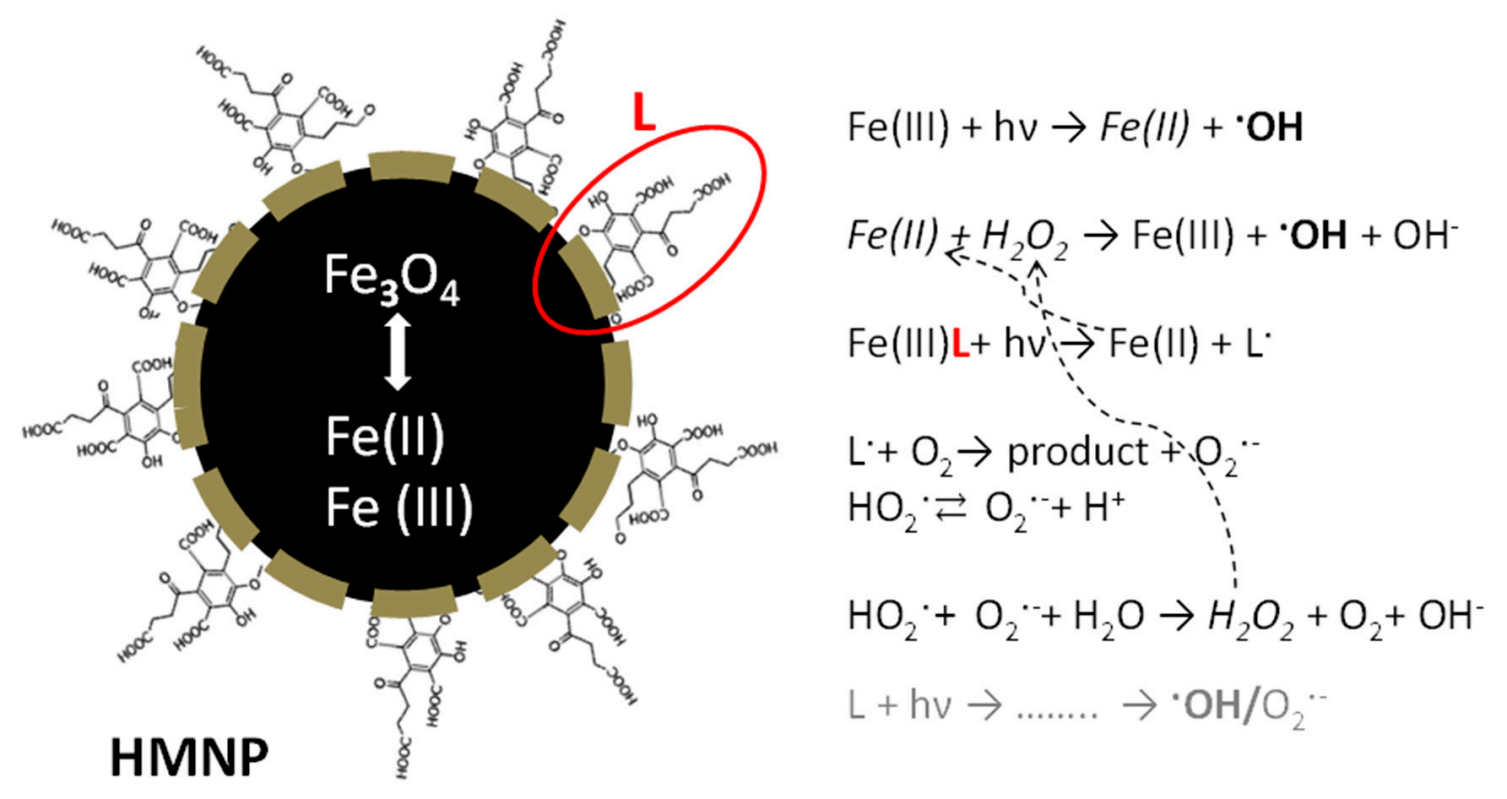
Schematic representation summarizing the main reactions occurring in the investigated system.

Scheme 2 summarizes the hypothesized mechanism yielding the production of the reactive species (mainly hydroxyl radicals) responsible for caffeine degradation.

The pH decrease upon the HMNPs’ addition, together with a not negligible iron ion released in solution, could contribute to the explanation for the decrement in caffeine abatement efficiency observed in the 2nd cycle, when MH0.2 was reused. Indeed, in the first cycle operated at pH = 4.5, both heterogeneous and homogeneous processes could occur. On the other hand, when MH0.2 were recovered and re-suspended, a slightly higher and less favorable pH (ca. 5.8–6.0) was observed, allowing for the hypothesis that in the second cycle the hydroxo-phase was already eliminated and only the heterogeneous photo-Fenton reaction took place. Even if the efficiency in the second cycle was lower, it is worth mentioning that the photo-Fenton-like reaction was still able to degrade caffeine efficiently, and in much less drastic conditions, since the working pH in the following experiment (i.e., in the absence of Fe(III) hydrolysis) was near the neutrality and this situation, together with the possibility of recovering the material thanks to its magnetism, makes the process potentially with no impact on the environment.

## 5. Conclusions

The use of HMNPs clearly evidenced the possibility of attaining pollutant degradation working at pH values closer to neutrality, overcoming the limit of pH = 2.8 characteristic of the homogeneous photo-Fenton process. Moreover, the added value of the organic coverage of the nanoparticles was demonstrated, thus allowing for the possibility to exploit organic waste as source of auxiliaries in environmental processes. Nevertheless, further investigation is needed to better understand the reaction, and in particular, to assess the capability of BBS-Cs to complex iron, in either Fe(II) or Fe(III) form.

Lastly, the good preliminary results obtained when reusing the nanoparticles encourage further studies in the direction of scaling-up the process to pilot plant for real application purposes.

## Figures and Tables

**Table 1:** Fe(II) and Fe(III) concentrations in water at different pH values from MH1.0 (200 mg·L^−1^) suspension after 12 h.

pH	Fe(II) (mg·L^−1^)	Fe(III) (mg·L^−1^)	Fe Tot. (mg·L^−1^)
3.0	0.837	0.153	0.993
5.0	0.036	0.014	0.049
7.0	0.137	0.051	0.189
